# Greater sensitivity of the circadian system of women to bright light, but not dim‐to‐moderate light

**DOI:** 10.1111/jpi.12936

**Published:** 2024-01-31

**Authors:** Parisa Vidafar, Elise M. McGlashan, Angus C. Burns, Clare Anderson, Ari Shechter, Steven W. Lockley, Andrew J. K. Phillips, Sean W. Cain

**Affiliations:** ^1^ School of Psychological Sciences and Turner Institute for Brain and Mental Health Monash University Clayton Victoria Australia; ^2^ Faculty of Medicine and Health, Central Clinical School The University of Sydney Sydney New South Wales Australia; ^3^ Australian Research Council Centre of Excellence for Children and Families over the Life Course Canberra Australian Capital Territory Australia; ^4^ Melbourne School of Psychological Sciences University of Melbourne Parkville Victoria Australia; ^5^ Department of Medicine Columbia University Irving Medical Center New York New York USA; ^6^ Departments of Medicine and Neurology, Division of Sleep and Circadian Disorders Brigham and Women's Hospital Boston Massachusetts USA; ^7^ Division of Sleep Medicine Harvard Medical School Boston Massachusetts USA; ^8^ Department of Clinical and Experimental Medicine, Faculty of Health and Medical Sciences Surrey Sleep Research Centre, University of Surrey Guildford Surrey UK

**Keywords:** evening light, gender differences, hormones, light sensitivity, melatonin suppression, menstrual phase, sex differences

## Abstract

Women typically sleep and wake earlier than men and have been shown to have earlier circadian timing relative to the light/dark cycle that synchronizes the clock. A potential mechanism for earlier timing in women is an altered response of the circadian system to evening light. We characterized individual‐level dose–response curves for light‐induced melatonin suppression using a within‐subjects protocol. Fifty‐six participants (29 women, 27 men; aged 18–30 years) were exposed to a range of light illuminances (10, 30, 50, 100, 200, 400, and 2000 lux) using melatonin suppression relative to a dim control (<1 lux) as a marker of light sensitivity. Women were free from hormonal contraception. To examine the potential influence of sex hormones, estradiol and progesterone was examined in women and testosterone was examined in a subset of men. Menstrual phase was monitored using self‐reports and estradiol and progesterone levels. Women exhibited significantly greater melatonin suppression than men under the 400‐lux and 2000‐lux conditions, but not under lower light conditions (10–200 lux). Light sensitivity did not differ by menstrual phase, nor was it associated with levels of estradiol, progesterone, or testosterone, suggesting the sex differences in light sensitivity were not acutely driven by circulating levels of sex hormones. These results suggest that sex differences in circadian timing are not due to differences in the response to dim/moderate light exposures typically experienced in the evening. The finding of increased bright light sensitivity in women suggests that sex differences in circadian timing could plausibly instead be driven by a greater sensitivity to phase‐advancing effects of bright morning light.

## 1 INTRODUCTION

Women tend to be more morning‐type than men, sleeping and waking at an earlier time.^1^, ^2^ This earlier timing in women appears to be due in part to having a faster circadian clock^3^ (shorter intrinsic circadian period), something also demonstrated in non‐human animals.^4^, ^5^

However, the size of the sex difference in circadian period in humans is modest (6 min, or 0.1 h, compared to a population range of ~1.5 h),^3^ suggesting other differences in circadian physiology likely contribute to differences in timing. One potential mechanism that could contribute to sex differences in time of day preference is the response of the circadian system to light.^6^ Sex differences may also explain some of the large interindividual differences in circadian light sensitivity.^7^

In humans, the structure of the suprachiasmatic nucleus (SCN; the core circadian clock) is sexually dimorphic. Analysis of postmortem tissue has shown that the shape of the SCN differs between women and men (elongated in women vs. spherical in men), though the overall volume, cell density, number of cells, and diameter of cell nuclei are similar.^8^, ^9^ An analysis of substructures in the SCN showed that the most striking sex differences were in vasoactive intestinal polypeptide (VIP) neurons. Clear sex differences in the number of VIP neurons in the SCN developed by the age of 10, with girls and women having half the number of VIP cells of boys and men.^10^, ^11^ Women continue to have fewer VIP cells until the age of 40, when men show a decline in VIP cells.^12^ Since VIP is a key modulator of light input to the circadian clock,^13^ these characteristics could translate to sex differences in circadian light sensitivity.

Three studies have examined potential sex differences in the sensitivity of the circadian system to light in humans using melatonin suppression as the outcome with inconsistent findings. An early small study (five men and five women) found no sex differences in melatonin suppression over a range of bright light levels from 1000 to 2500 lux.^14^ Another small study (six men and six women) found that women had greater melatonin suppression to bright light (2000 lux).^15^ A larger study found no sex differences to moderate light levels (200 and 500 lux).^16^ In this study, 21 women and 22 men were compared at 200 lux, but only four men and seven women at 500 lux. The light exposure procedures and methods of calculating suppression differed across these studies. Furthermore, none of these previous studies compared responses between luteal and follicular menstrual phases. One small within‐subject study in six women found no effect of menstrual phase on melatonin suppression to a moderate (200 lux) 1‐h light exposure.^17^

Here, we investigated sex and menstrual phase differences in melatonin suppression to a broad range of evening light exposure levels (10–2000 lux) in the same individuals. To examine potential sex differences in light sensitivity, we generated individual‐level dose–response curves to light by systematically varying light exposures within individuals over a 6–7‐week protocol in 29 women and 27 men. Using these highly controlled light‐exposure techniques, we found that women were more sensitive only to the brightest light levels, with no sex differences seen in dim/moderate light (200 lux and below).

## 2 MATERIALS AND METHOD

### 2.1 Participants

Sixty‐one participants were enrolled. Four were excluded based on actigraphy, and one was excluded due to scheduling conflicts. A total of 56 healthy young men and women (29 women, 27 men; 18–30 years; all of European ancestry to control for genetic testing) completed the study. These data were previously used to describe large interindividual differences in circadian light sensitivity.^7^ One woman was excluded from the analysis as her dim light melatonin onset (DLMO) could not be determined. Data from the remaining 55 participants (28 women, 27 men; 20.8 ± 2.6 years) were analyzed. Participants were free from physical and mental illnesses and did not use recreational drugs, medication (including hormonal contraceptives) or nicotine during the study. Body mass index (BMI) was within the range 18–30 kg/m^2^. To ensure stable entrainment to their fixed, self‐selected, 8:16‐h sleep:wake schedules before and during the experiment, only individuals who had not traveled across more than one time zone within 3 months from admission or engaged in shift work over the past year were enrolled in the study.

Participants were asked to not consume caffeine and alcohol for 1 week before their first admission, and throughout the testing period. Abstinence from recreational drugs and medications for 3 weeks before admission, and throughout their enrollment was required and confirmed before each test session via urine toxicology (SureStep Single Drug Cassette, Innovacon Inc., San Diego, USA). Breath tests were conducted at each session to verify abstinence from alcohol (Enforcer 2 Breathalyzer, Alcolimit, New South Wales, Australia). Women were also tested for pregnancy at each visit (Xcel One‐Step Pregnancy Test, Princeton BioMeditech, New Jersey, USA).

### 2.2 Study design and protocol

Participants completed a total of 6 or 7 weekly in‐lab test sessions, which commenced 5 h before scheduled (self‐selected) bedtime and concluded 1 h after scheduled bedtime. During each session, a controlled light exposure was administered from 4 h before habitual bedtime until 1 h after habitual bedtime. The first of these sessions was always a dim control (<1 lux), with subsequent sessions of experimental light exposures of varying intensities (10, 30, 50, 100, 200, 400, and 2000 lux). Participants were randomized to one of six light exposure orders: (a) 100, 200, 10, 2000, and 400 lux (*n* = 10; six women, four men); (b) 10, 100, 400, 200, and 2000 lux (*n* = 12; six women, six men); (c) 2000, 400, 200, 100, and 10 lux (*n* = 11; five women, six men); (d) 200, 400, 100, 10, 30, and 50 lux (*n* = 10; five women, five men); (e) 100, 30, 200, 50, 400, and 10 lux (*n* = 3; two women, one man); and (f) 10, 50, 30, 200, 100, and 400 lux (*n* = 10; five women, five men). A total of 33 participants were randomly assigned to orders (a–c) and 23 were randomly assigned to orders (d–f). Contrast analyses for DLMO and phase angle confirmed no differences between orders (*p*_all_ > .05). All procedures received ethical approval from the Monash University Human Research Ethics Committee, and participants gave written informed consent before enrollment in the study. Participants were remunerated for their participation.

#### 2.2.1 At‐home sleep monitoring

Participants were required to maintain a fixed 8:16‐h, sleep:wake, dark:light schedule for at least 1 week before each lab admission (19.36 ± 7.85 days). Bed and wake times were self‐selected and adherence to this regimented schedule was assessed on a weekly basis via sleep diaries and actigraphy. Naps were not permitted and a deviation greater than 30 min from the agreed upon bedtime on more than one night per week resulted in exclusion from the study (*n* = 3). This was verified by wrist‐worn actigraphy (Actiwatch‐L/2/Plus/Pro, Philips Respironics, Koninklijke, Netherlands) and sleep diaries throughout the study protocol. Women noted menses onset and offset in their sleep diaries throughout the duration of the study protocol.

#### 2.2.2 In‐laboratory sessions

A single LED light source with a peak of 451 nm and a correlated color temperature (CCT) of 4289 Kelvin located at the back of the suite (out of the participants’ line of vision) was used to create the dim condition (<1 lux) for the first lab session. Experimental light exposures (10–2000 lux) were achieved using Philips 4100 Kelvin bulbs (Master TL5 HE 28 W/840, Philips, Eindhoven, Netherlands). These bulbs provided a broad spectrum of light with a peak of 545 nm and a CCT of 3968 Kelvin. To achieve an even room distribution under the dimmer conditions (10, 30, and 50 lux), one‐stop neutral density filters were placed over the ceiling mounted bulbs (LEE 209 Neutral Density Filter, Lightmoves, Victoria, Australia).

Participants alternated between 10‐min of fixed gaze and 10‐min of free gaze throughout the 5‐h light exposures. During fixed gaze, participants were instructed to direct their gaze toward a specified point (measured to match the intensity of each light exposure in the vertical plane at the eye) on the wall they were facing. This was to ensure intra‐ and inter‐ individual stability of the light exposures. Light illuminance was measured hourly using a lux meter (Tektronix J17 Luma Color, Oregon, USA) at the level of both eyes at the vertical angle of gaze during each session. The effective daylight illuminances for human photoreceptors in the retina across the light exposure conditions in our study according to the CIE tool box,^18^ and the average Daylight Effective Ratio (DER) across light levels are shown in Table 1.

**Table 1:** Effective daylight illuminances for human photoreceptors, irradiance, and photopic illuminance across light exposure conditions.

	Irradiance	Photopic	S‐cone‐opic	M‐cone‐opic	L‐cone‐opic	Rhodopic	Melanopic
~10 Lux	4.40	10.83	5.52	9.42	10.61	6.64	5.52
~30 Lux	10.16	30.91	14.14	26.41	30.26	17.64	14.19
~50 Lux	15.97	50.14	22.08	42.85	49.03	28.49	22.79
~100 Lux	31.05	100.63	47.29	86.97	98.27	59.40	48.24
~200 lux	61.67	200.20	102.91	174.25	195.50	121.25	99.76
~400 lux	124.31	403.43	0.53	0.87	0.98	0.62	0.51
~2000 lux	586.97	2036.70	1093.25	1772.94	2007.39	1296.68	1106.21
**Mean DER**			0.49	0.87	0.98	0.60	0.49

*Note*: Daylight effective ratio (DER) is presented as the mean across measurements, and illuminances and irradiance are reported for power between 380 nm and 760 nm.

### 2.3 Salivary melatonin assays

Saliva samples were collected hourly during the 5‐h light exposure (total of six samples per session) using salivettes (Salimetrics, Inc., California, USA) and participants were instructed to abstain from water and food and remain seated for at least 20 min before each sample was collected. Samples were centrifuged at 2500 rpm for 5 min (Centrifuge 5702 R, Eppendorf, San Diego, USA), before being placed in a −20°C freezer for storage until assay. Melatonin was assayed in saliva in duplicate (200 μl) at the Adelaide Research Assay Facility, Robinson Research Institute, University of Adelaide by double antibody radioimmunoassay (RKDSM‐2), using reagents supplied by Buhlmann Laboratories AG, Schönenbuch, Switzerland. This assay is based on the Kennaway G280 anti‐melatonin antibody,^19^ uses [125I]2‐iodomelatonin as the radioligand. The sensitivity of the assay was 4.3 pM. The intra‐assay coefficient of variation of the assays was 7.0%. The inter‐assay coefficient of variation of the assays was <17% for quality controls containing 8.5–16.4 pM, and <17% for quality controls containing 77.2–192.9 pM melatonin.

### 2.4 Salivary sex hormone assays

During each test session, a single saliva sample was collected approximately 3 h before scheduled bedtime for each woman to assay for female sex hormones and objectively verify menstrual phase. Salivary estradiol, progesterone, and testosterone were measured using commercially available ELISA kits (Salimetrics, USA). Menstrual phase was estimated using the salivary estradiol and progesterone measures and self‐reports of menses onset. Before enrollment, women provided information about their menstrual cycles during the phone screen and in‐person screening interview. This information included dates for menses onset and offset for their most recent menstrual cycle, duration of menses for their most recent menstrual cycle (4.44 ± 1.15 days), and average number of days in their typical menstrual cycle (28.80 ± 3.58 days). Once admitted to the study, women were asked to note menses onset and offset dates on their sleep diaries, which were checked every week. The forward‐counting method^20^ was used to estimate menstrual phase from the self‐reports. In this method, the number of days from menses onset are counted prospectively, with the first 14 days after menses onset considered as the follicular phase and the days following ovulation considered as the luteal phase. We confirmed menstrual phase using salivary progesterone and estradiol levels plotted across the six to seven test sessions. Menstrual phase was determined by plotting the absolute values for progesterone and estradiol to create a hormone profile for each participant over the study duration. Ovulation was then determined for each participant using the lowest concentration of progesterone preceding the peak progesterone and confirmed via visual inspection of their hormone profiles. Once ovulation was determined, menses was determined using the lowest concentration of progesterone and estradiol. Three broad phases were determined as: (1) menses (onset and offset of bleeding); follicular (post menses until ovulation); and luteal (post‐ovulation until menses onset). In cases where there was discrepancy of menses onset/offset between objective measures and self‐reports, objective measures were used. This occurred in ~34% of our sample.

### 2.5 Data analysis

Dim light melatonin onset (DLMO) during the <1 lux condition was determined using an absolute threshold of 4 pg/ml. Interpolation was used to determine the time at which DLMO was achieved by calculating the rate of change between the last sample below the threshold, and the first sample to reach or exceed this threshold. Phase angle to bedtime was then determined by calculating the difference between DLMO time and each individuals’ scheduled bedtime. As light exposures were administered based on scheduled bedtime and not circadian clock time, we calculated total melatonin suppression from the <1 lux DLMO to the end of the light exposure in each participant, using the area under the curve (AUC; trapezoidal method). The melatonin suppression for each experimental light condition was quantified as the percentage change in the AUC of the salivary melatonin profile relative to the dim light condition (<1 lux). Cleaning rules were applied to the suppression values, as in previous work^7^: individual‐level dose–response curves were assumed to be monotonically increasing, with a 20% tolerance for experimental error and intraindividual variability. Suppression values that were more than 20% less than the suppression values at any lower light levels were excluded.

As in previous work,^7^ dose–response curves were fitted using a two‐parameter logistic equation, with the two parameters representing (i) the light dose required for 50% melatonin suppression (ED50) and (ii) a parameter determining the sigmoid width. These fits were performed at both individual and group levels (men vs. women, luteal vs. follicular phase). To ensure convergence of the fit, suppression values were bounded to a minimum of 0.1% and a maximum of 99.9%. Individual‐level estimates of the ED50 were considered reliable if the 95% CI for the individual‐level fit of the ED50 spanned <1 log10‐unit (i.e., a factor of 10). Fitting was performed using the function nlinfit in MATLAB R2021a, with 95% confidence intervals for the fitted parameters obtained using the function nlparci.

Melatonin suppression values were compared at each light level between groups (men vs. women; luteal vs. follicular) using independent samples *t*‐tests. As secondary analysis, suppression values were also compared for each time point during light exposure at each light level using independent samples *t*‐tests. Reliable individual‐level ED50 values were compared between groups (men vs. women; luteal vs. follicular) using independent samples *t*‐tests. To investigate which sex‐related factors predicted melatonin suppression, tobit regression models were used to incorporate multiple predictors. A tobit model was selected due to both lower and upper bounds on the continuous‐valued dependent variable (melatonin suppression). Separate tobit models were run with the primary predictor variable(s) being: (i) sex, (ii) menstrual phase (women only), (iii) estradiol and progesterone levels (women only), and (iv) testosterone (men only, using the mean of the two testosterone samples). For each analysis, both an unadjusted model and an adjusted model were run. The unadjusted model included the primary predictor and light. The adjusted model also included age, sex (where applicable), and the phase angle of habitual bedtime to DLMO (to adjust for the circadian phase at which samples were collected). Unstandardized coefficients (estimate±SE) and *p*‐values are reported for each model for the primary predictors. Finally, in men we tested for a linear correlation between testosterone values and the corresponding melatonin suppression response at the 100‐lux and 200‐lux conditions where testosterone was measured.

## 3 RESULTS

### 3.1 Women were more sensitive to bright light than men

**Figure 1:**
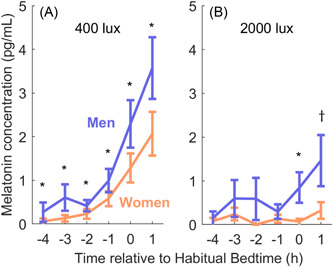
Melatonin levels during the 400‐lux and 2000‐lux conditions in men versus women. Average melatonin levels are plotted (mean ± SEM) for the women (orange) and men (blue) at each hourly time point during the (A) 400‐lux and (B) 2000‐lux light conditions. Significance is shown for independent samples *t*‐tests (*p < .05, †*p* < .10).

**Figure 2:**
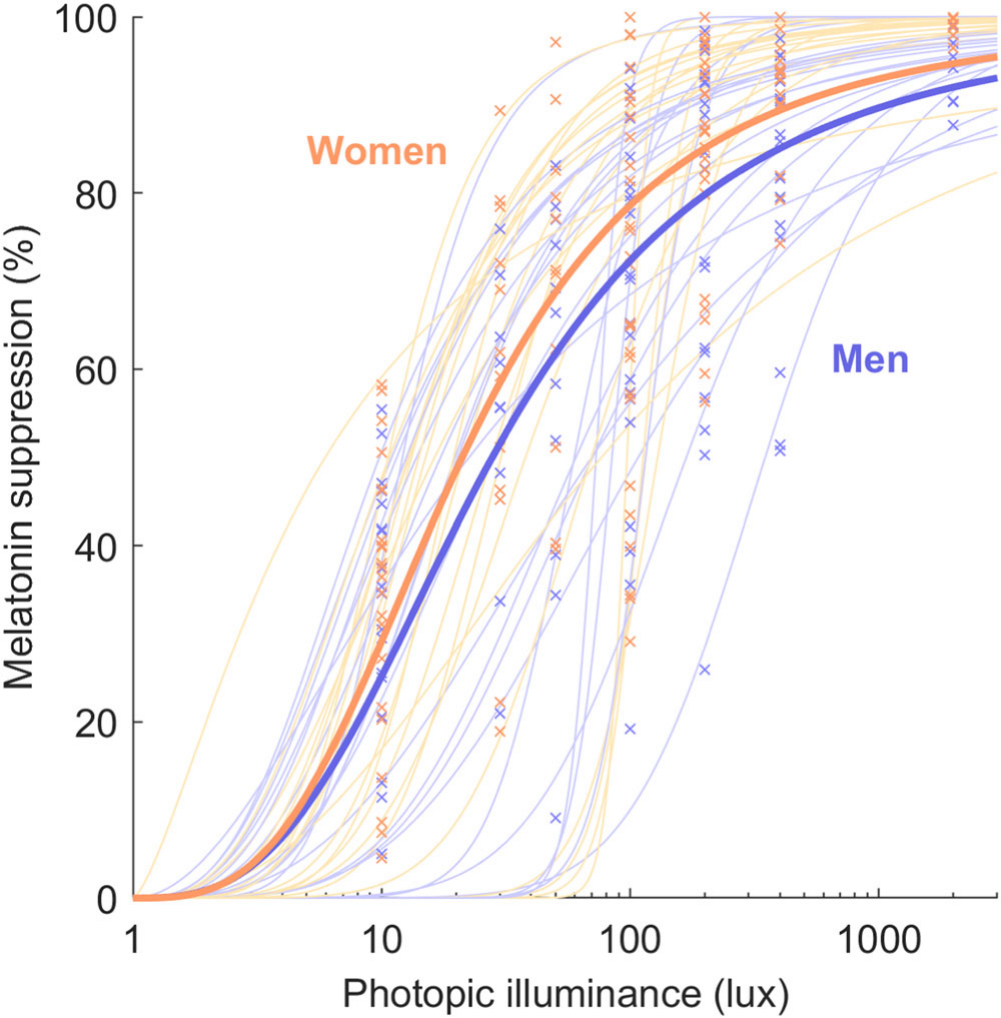
Percentage melatonin suppression under each light condition for men versus women. Suppression values are given as a percentage relative to dim light <1 lux conditions. Individual data points are shown as crosses for the women (orange) and men (blue). Group‐level dose response curves for women and men are plotted as thick orange and blue lines, respectively. Individual‐level dose–response curves are plotted as thin lines. A logarithmic x‐axis is used.

We investigated melatonin suppression responses at each light level from 10 to 2000 lux. We found that women were significantly more sensitive than men to the two brightest stimuli: the 400‐lux stimulus (93.7 ± 9.6% suppression in *n* = 28 women vs. 83.2 ± 18.7% suppression in *n* = 27 men, *p* = .01) and the 2000‐lux stimulus (99.5 ± 1.0% suppression in *n* = 17 women vs. 96.9 ± 4.3% suppression in *n* = 16 men, *p* = .03). These sex differences were reflected in lower absolute values of melatonin for women at all timepoints under the 400‐lux condition and one timepoint under the 2000‐lux condition, shown in Figure 1. However, there were no significant differences between men and women detected at any of the lower light levels we tested, ranging from 10 to 200 lux (all *p* > .16), and there were no significant differences in the parameters of the overall dose–response curves (Figure 2) for the 10–2000 lux data. Though there was a trend towards higher sensitivity in women, there were no significant differences in either the group‐level ED50 (95% confidence intervals of 10^[1.25,1.40] for women and 10^[1.35,1.54] for men) or sigmoid width parameter (95% confidence intervals of [2.65,3.66] for women and [2.44,3.48] for men). Consistent with this finding, we also found no significant difference in the individual‐level fits of the ED50 (light level required for 50% melatonin suppression) between men and women (*p* = .70). Using a tobit regression model with light level and sex as predictors, we found that sex was not a significant predictor of melatonin suppression in the unadjusted model (*β* = .09 ± .06, *p* = .14), but was significant when adjusted for age and phase angle (*β* = .13 ± .06, *p* = .04), whereas the light level was a highly significant predictor of suppression (*p* < .0001). These findings together indicate a strong similarity between women and men in the response to light levels up to 200 lux, with a pronounced difference in sensitivity only under bright conditions.

### 3.2 Menstrual phase did not relate to light sensitivity

**Figure 3:**
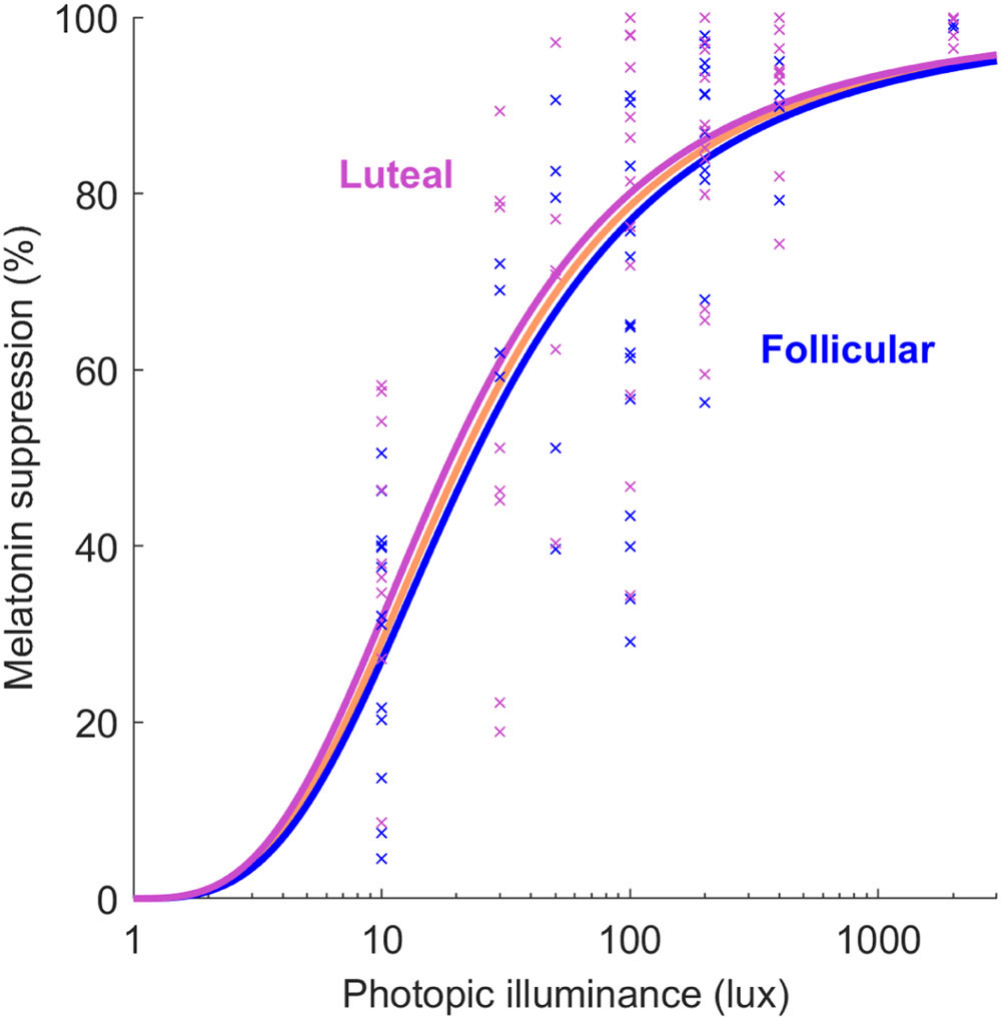
Percentage melatonin suppression under each light condition for luteal versus follicular phases in women. Suppression values are given as a percentage relative to dim light (<1 lux) conditions. Individual data points are shown as crosses for the luteal (pink) and follicular phases (blue). Group‐level dose response curves for luteal and follicular phases are plotted as thick pink and blue lines, respectively. The group‐level fit for all of the women (combining menstrual phases) is shown for reference as the think orange line. A logarithmic x‐axis is used.

In a stratified analysis of the female participants, we investigated whether menstrual phase (follicular vs. luteal) at the time of light exposure was a predictor of light sensitivity. Both menstrual phases were represented at each light level (<1 lux: 18 luteal, 11 follicular; 10 lux: 13 luteal, 16 follicular; 30 lux: 8 luteal, 4 follicular, 50 lux: 6 luteal, 6 follicular; 100 lux: 14 luteal, 15 follicular; 200 lux: 13 luteal, 16 follicular; 400 lux: 18 luteal, 11 follicular; 2000 lux: 9 luteal, 8 follicular). There were no significant differences between menstrual phases in the parameters of the overall dose–response curves (Figure 3) for the 10–2000 lux data, either for ED50 (95% confidence intervals of 10^[1.26,1.48] for follicular and 10^[1.16,1.40] for luteal) or sigmoid width parameter (95% confidence intervals of [2.47,3.87] for follicular and [2.36,3.86] for luteal). Using a tobit regression model with light level and menstrual phase as predictors, we found that menstrual phase (follicular vs. luteal) was not a significant predictor of melatonin suppression in either the unadjusted (*β* = −.01 ± .06, *p* = .83) or adjusted models (*β* = −.02 ± .04, *p* = .70), whereas light level was a highly significant predictor (*p* < .001). Comparing melatonin suppression for each light level from 10 to 2000 lux, we also found no significant differences in melatonin suppression between women in the follicular phase and women in the luteal phase (all comparisons *p* > .08).

### 3.3 Sex hormone levels did not relate to light sensitivity

We further investigated whether sex hormones accounted for individual differences in the melatonin suppression response. First, we investigated in women participants (*n* = 28) whether progesterone or estradiol levels collected at the time of each light exposure condition were related to light sensitivity. Using a tobit regression model with light level, progesterone, and estradiol as predictors, we found that neither progesterone (*β* = −.0003 ± .0003, *p* = .34 unadjusted; *β* = −.00001 ± .00024, *p* = .96 adjusted) nor estradiol (*β* = .02 ± .03, *p* = .70 unadjusted; *β* = .09 ± .06, *p* = .59 adjusted) were significant predictors of melatonin suppression, whereas light level was a highly significant predictor (*p* < .001). Next, we investigated in a subset of the male participants (*n* = 16) whether testosterone levels were related to light sensitivity. In each participant, testosterone levels were measured twice: at the time of the 100‐lux and 200‐lux light exposures. We found no significant association between melatonin suppression and the corresponding testosterone measurement at either the 100‐lux (*r* = .21, *p* = .44) or 200‐lux (*r* = −.20, *p* = .47) exposures. There was also no significant association between each individual's average testosterone level and their individual‐level ED50 from their dose–response curve to the 10–2000 lux data (*r* = .07, *p* = .80). Using a tobit regression model with light level and average testosterone level as predictors, we found that testosterone level was not a significant predictor of melatonin suppression (*β* = −.0001 ± .0015, *p* = .93 unadjusted; *β* = .0002 ± .0015, *p* = .89 adjusted for age and habitual time in bed), whereas light level was a highly significant predictor (*p* < .001).

## 4 DISCUSSION

We found that women are more sensitive to bright evening light than men. Women demonstrated significantly greater melatonin suppression under 400‐lux and 2000‐lux conditions. At dim‐to‐moderate light levels (200 lux and below), men and women had similar responses. Considering the entire dose–response curves of women and men, we found no difference in the light level needed to have melatonin suppressed by half, due to this response occurring in the dim‐to‐moderate range. In women, menstrual phase did not influence light sensitivity at any light level, and melatonin suppression was unrelated to estrogen and progesterone levels. Similarly, in men, testosterone levels were unrelated to melatonin suppression at all light levels.

Our results clarify discrepancies between previous studies examining sex differences in melatonin suppression to bright light. In the earliest study comparing melatonin levels during a light exposure in men and women (five men and five women), no sex difference was seen^14^; however, there were also no significant differences in suppression observed between light levels 1000–2500 lux (and this was not due to a ceiling effect at lower levels). This prior study did not examine melatonin suppression directly (a comparison of melatonin levels in the dark vs. light), but rather melatonin levels under different light levels. As there are large interindividual differences in melatonin levels,^21^, ^22^, ^23^ without a dark control, little can be concluded on light sensitivity as the level of suppression is unknown. A similarly sized later study (six men and six women) examined melatonin suppression, comparing melatonin levels in the dark versus 2000 lux.^15^ A large sex difference was found, with men showing less than half the suppression seen in women across the 1‐h light exposure. Our results, also based on a comparison of individuals’ melatonin levels in the dark versus light, agree with the latter study showing sex differences under bright light.

The largest previous study of sex differences in light sensitivity by Nathan et al.^16^ examined melatonin suppression to light in women and men at 200 lux and 500 lux, showing no significant differences at either level. Though this seemingly contradicts our observed differences at 400 lux, a closer examination of the previous study suggests similar findings. Nathan et al. examined melatonin suppression to 200 lux in 21 women and 22 men, whereas at 500 lux only four men and seven women were examined. Thus, the study may not have been powered to detect the difference at 500 lux. We note that the data presented in that study do suggest a sex difference in suppression at 500 lux. Women appeared to have a maximum suppression (calculated as the lowest levels during light compared to levels immediately before light onset) that was ~40% greater than men. In our study, we measured melatonin suppression in 29 women and 27 men at 400 lux and found a significant difference. Taken together, our results clarify previous studies, demonstrating that at bright light levels, the circadian system of women is more sensitive to light than men, with responses at lower levels showing no meaningful sex differences.

We found no effects of menstrual phase or estrogen and progesterone levels on light sensitivity in women. This finding is consistent with previous work showing no effect of menstrual phase on suppression to 200 lux of light.^17^ Our results are also consistent with studies showing no effect of menstrual phase on melatonin timing,^24^, ^25^ since variability in light sensitivity with menstrual phase would lead to variability in the phase of entrainment. Assuming similar self‐selected light exposure patterns across menstrual phases in women, the stability in circadian phase across the menstrual cycle is consistent with light sensitivity being unaffected by sex hormone levels. Though we found no effects of female sex hormones on light sensitivity, it is important to note that we studied a healthy population. Furthermore, our method for measuring salivary estradiol and progesterone at a single time point once a week for 6–7 weeks using commercially available ELISA kits cannot capture the full range of hormonal fluctuations across the menstrual cycle for each woman in our study. As such, our results showing no association between the menstrual cycle and light sensitivity should be interpreted with caution. Progesterone and estrogen receptors have been identified in the SCN of humans.^26^ Both men and women have estrogen and progesterone receptors in the SCN, with women having significantly higher levels of the estrogen receptor alpha subtype. Increased sensitivity to light‐induced melatonin suppression has been demonstrated in Premenstrual Dysphoric Disorder (PMDD).^27^ In PMDD, hormone levels are not necessarily altered, although sensitivity to them is increased.^28^ Thus, it is likely that female sex hormones can influence light sensitivity under some circumstances. Indeed, gonadectomy in female mice has been shown to influence circadian light sensitivity.^29^ Future work examining changes in light sensitivity in women on hormonal contraception or hormone replacement therapy could reveal important effects on the circadian system.

We examined testosterone levels in a subset of men and found no relation of testosterone levels and melatonin suppression to any light level. Androgen receptors are present in the SCN of humans, with greater expression in men.^30^ Non‐human studies localizing receptors to subregions of the SCN demonstrate a greater expression in core of the SCN,^31^ suggesting a potential role in modulating light input. This is supported by the finding that, in mice, castration reduces the expression of FOS in the SCN after phase‐delaying or phase‐advancing light pulses, an effect reversed by hormone replacement.^31^ It is possible that androgens have less of an effect on circadian light sensitivity in humans, though our lack of an effect may be due to a limited range in testosterone levels. Our sample of healthy young men had a range of 57.30–240.13 pg/ml. An effect of testosterone on circadian light sensitivity in humans might be seen if more extreme values were examined.

Our finding of sex differences in circadian light sensitivity only at bright light levels implies that no sex difference would be expected for typical light exposure within homes in the evening, as these high levels would rarely be encounted.^32^ However, shift workers in some settings likely experience brighter night time light exposure, which might contribute to the poorer sex difference tolerance in women.^33^, ^34^ Furthermore, although we investigated an evening light exposure, it is plausible that these differences in sensitivity apply to the effects of light on the circadian clock at other circadian phases. Greater sensitivity to daylight in women partly explains earlier timing in women than men. The greater response of women to bright light implies that the circadian system of women may be more sensitive to the effects of daylight on the circadian system. Bright light exposure in the morning induces phase advances, while bright light exposure in the day can boost circadian amplitude.^35^, ^36^ In our study, we examined only melatonin suppression and not phase shifting, two responses to light that are dissociable under certain conditions.^37^ A direct examination of sex differences in phase shifting is needed to confirm this potential effect of increased responsiveness to bright light in women.

In summary, our results reveal that healthy men and women have similar circadian responses to dim/moderate light exposure in the evening, at levels that would typically be experienced in a home setting. However, women have a higher sensitivity to the effects of bright light on the circadian system. Women's increased sensitivity to bright light might have important implications for sex differences in circadian timing.

## AUTHOR CONTRIBUTIONS

Steven W. Lockley and Sean W. Cain conceived and designed the study; Parisa Vidafar, Elise M. McGlashan, and Angus C. Burns performed the research and acquired the data; Parisa Vidafar, Angus C. Burns, Steven W. Lockley, Andrew J. K. Phillips, and Sean W. Cain analyzed and interpreted the data; Parisa Vidafar, Andrew J. K. Phillips, and Sean W. Cain drafted the manuscript; Parisa Vidafar, Elise M. McGlashan, Angus C. Burns, Ari Shechter, Clare Anderson, Steven W. Lockley, Andrew J. K. Phillips, and Sean W. Cain reviewed and edited the manuscript. All authors have read and approved of the submitted manuscript.

## Data Availability

The data that support the findings of this study are available on request from the corresponding author. The data are not publicly available due to privacy or ethical restrictions. Data made available on request due to privacy/ethical restrictions.
